# Diagnostic value of microRNA-155 in active tuberculosis: A systematic review and meta-analysis

**DOI:** 10.1097/MD.0000000000027869

**Published:** 2021-11-19

**Authors:** Xiaoyan Li, Jie He, Guodong Wang, Jian Sun

**Affiliations:** a Department of Endocrinology, Clinical Medical College and The First Affiliated Hospital of Chengdu Medical College, Chengdu, Sichuan, PR China; b Department of Pulmonary and Critical Care Medicine, Clinical Medical College and The First Affiliated Hospital of Chengdu Medical College, Chengdu, Sichuan, PR China; c Department of Pathology, Mouping District Hospital of Traditional Chinese Medicine, Yantai, Shandong, PR China.

**Keywords:** biomarker, diagnosis, meta-analysis, microRNA-155, tuberculosis

## Abstract

**Background:**

Tuberculosis (TB) is a preventable and treatable disease, but the increased mortality and morbidity associated with TB continues to be a leading cause of death globally. MicroRNA (miRNA)-155 has been recognized as a marker of many lung diseases. However, the effectiveness of this marker for diagnosing TB remains unclear.

**Methods:**

A detailed search (updated on February 6, 2021) of literature published in the Wanfang database, EMBASE, PubMed, CNKI, and Cochrane Library was conducted to identify eligible studies suitable for inclusion in the current research. The positive likelihood ratio, negative likelihood ratio, specificity, area under the curve, sensitivity, and diagnostic odds ratio were used to investigate the diagnostic potential of miRNA-155.

**Results:**

A total of 122 studies related to active TB, which completely complied with the inclusion and exclusion criteria of our meta-analysis, were included. The overall results suggested a moderately high diagnostic accuracy and efficacy of miRNA-155, with a specificity of 0.85 (95% confidence interval = 0.77–0.91) and sensitivity of 0.87 (95% confidence interval = 0.76–0.93). The result based on dysregulated status demonstrated that the upregulated group yielded better accuracy and efficacy than the downregulated group. Notably, the accuracy and efficacy of miRNA-155 in pediatric TB were higher than those in adult TB. The results showed that the accuracy and efficacy of miRNA-155 in children were higher than those in adults.

**Conclusion:**

The results of the meta-analysis suggested that miRNA-155 could serve as an effective biomarker for identifying active TB.

## 1 Introduction

Tuberculosis (TB) is a lethal infectious disease, transmitted by cough-propelled droplets carrying *Mycobacterium tuberculosis* (MTB).^[1]^ According to reports published by the World Health Organization, approximately 10 million new cases of TB were reported in 2018 worldwide, and more than 1.2 million people died of this disease and its related complications in the same year.^[2]^ Although people living with latent TB lack specific clinical symptoms, 10% of patients are at risk of developing active TB.^[3]^ Pulmonary TB causes many deaths globally, and its treatment is expensive.^[4]^ China accounts for more than 10% of the global incidence of pulmonary TB, which is a major public health issue.^[5]^ Although the prevalence of active pulmonary TB in China has decreased, the proportion of asymptomatic patients with active pulmonary TB has increased significantly.^[6]^

The routine methods for diagnosing active pulmonary TB include symptom assessment, radiological examination, and microbiological testing.^[7]^ Coughing is a common symptom of TB, but it is not specific to TB. In addition to other detection methods, radiological examinations should be conducted to test for TB. Although acid-fast staining has a high specificity (SPE), its sensitivity (SEN) is less than 30%.^[7]^ Tissue biopsy is an invasive test, and its accuracy is largely affected by the operator's clinical experience.^[8]^ The cost of GeneXpert equipment is high and hence, it is not suitable for use in small district hospital.^[9]^ The culture SEN of MTB is also not ideal, and the culture duration is lengthy.^[10]^ The laboratory biosafety level is high; thus, the early diagnosis of pulmonary TB is difficult.^[11,12]^ Therefore, devising a new, rapid and simple auxiliary diagnostic method to facilitate the early treatment of active pulmonary TB is the key to improving patient prognosis and reducing pulmonary TB complications.

MicroRNAs (miRNAs) are a class of small (≈18–24 nucleotides long), single-stranded, noncoding RNAs.^[13]^ Mature miRNAs effectively bind to the 3′-untranslated regions of target mRNAs, leading to their degradation or translational repression (ie, the regulation of gene expression). miRNAs play an important role in cell proliferation, apoptosis, cellular metabolism, and immunity.^[14]^ Due to their stability in the plasma and sputum and high SEN in measurements, miRNAs can be used as a molecular marker for many diseases.^[15]^ In addition, miRNAs can bind to various molecules in the plasma and participate in the maintenance of homeostasis through endocrine, paracrine, or autocrine systems. In recent years, miRNA-155 has been extensively studied for TB diagnosis. Kathirvel et al^[16]^ suggested that miRNA-155 could serve as a useful biomarker in active pulmonary TB, and that it could be an important part of the mechanism of pulmonary TB, especially in pediatric TB. Bonilla-Muro et al^[17]^ also showed that EsxA was predominantly involved in the overexpression of miRNA-155 in human monocyte-derived macrophages, which could affect its involvement in the immune mechanism of macrophages through miRNA imbalance.

Although miRNA-155 has been widely investigated in TB-related studies, the results of domestic and international studies on the miRNA-155-based diagnosis of active pulmonary TB are not consistent. This may be due to their small sample size, diverse sample sources, and different detection methods. The present study aimed to comprehensively evaluate the value of miRNA-155 in the diagnosis of active pulmonary TB through a meta-analysis and provide an evidence-based basis for further research and clinical application of miRNA-155.

## 2 Methods

### 2.1 Literature retrieval

Studies published in the Wanfang database, EMBASE, PubMed, CNKI, and Cochrane Library before February 6, 2021 were retrieved. To search through the published literature, the keywords search strategy was employed as follows: “microRNA-155”, “miRNA-155”, “MiR-155”, “tuberculosis”, “pulmonary tuberculosis”, “mycobacteria”, “TB”, and “PTB”. The bibliographic list of pertinent studies was also searched to retrieve supplementary reviews. The language of the selected studies was restricted to English or Chinese.

### 2.2 Exclusion and inclusion criteria

The exclusion criteria were as follows: poor or inadequate data provided in the literature; animal studies, and in vitro studies; and parts of published literature such as reviews, meta-analyses, case reports, letters, and conference abstracts. The inclusion criteria were as follows: sufficient data provided in the literature; retrospective studies, prospective studies, and randomized controlled trials; studies focusing on the diagnostic accuracy and efficacy (DAE) of miRNA-155 in patients with active TB; measurement of miRNA-155 expression in sputum and serum samples; and MTB culture and other diagnostic measurements, such as pathological examinations and MTB/rifampicin tests, with the former being the gold standard method of diagnosis. All analyses in this meta-analysis were based on previously published studies; consequently, no ethical approval or patient consent was required.

### 2.3 Ethical review

Ethical approval and patient consent were not required because the meta-analysis was based on published research.

### 2.4 Quality assessment and data extraction

Quality assessment of diagnostic accuracy studies-2 (QUADAS-2) was carried out to determine the study design and attributes of each included article.^[18]^ Two independent reviewers were tasked with performing a thorough evaluation of the study, during which possible disagreements in proceedings were discussed, and a unanimous decision was reached. Detailed information, such as the year of publication and country, false-positive, SPE, sample types, false-negative, author's first name, SEN, true-negative, and participant characteristics, was extracted from the available literature search. Patients were evaluated for 4 critical aspects, including applicability concerns and the risk of bias, using the QUADAS-2 tool.

### 2.5 Statistical analysis

Statistical analysis was performed using Review Manager 5.3 software (Cochrane Collaboration, Copenhagen, Denmark) and Stata 10.0 software (Stata Corporation, College Station, Taxas, USA). We analyzed the pooled positive likelihood ratio (PLR), SEN, negative likelihood ratio (NLR), SPE, diagnostic odds ratio (DOR), and the corresponding 95% confidence intervals (CIs) for the miRNA-155 study using a random-effects model. We also determined the SEN and SPE values of miRNA-155 in this meta-analysis using the summary receiver operating characteristic curve, where the area under the curve (AUC) was also calculated. Furthermore, SEN assessment and subgroup effect analysis (SEA) were employed to explore the potential sources of heterogeneity. Finally, publication bias was evaluated by using Deeks funnel plot asymmetry test.

## 3 Results

### 3.1 Characteristics of eligible studies and the selection process

**Figure 1:**
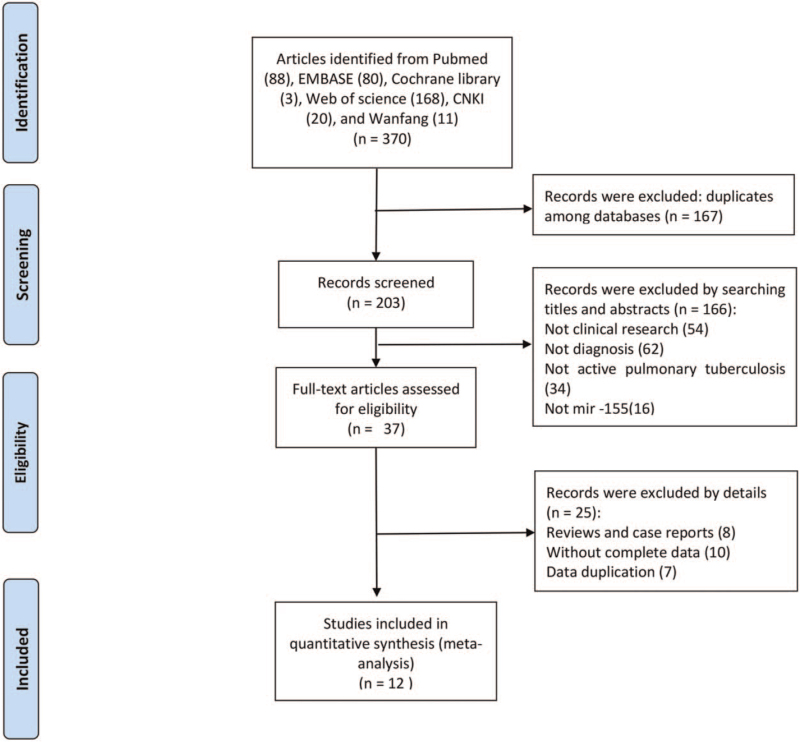
Flowchart of the article's selection process in the meta-analysis. CNKI = China National Knowledge Infrastructure.

**Figure 2:**
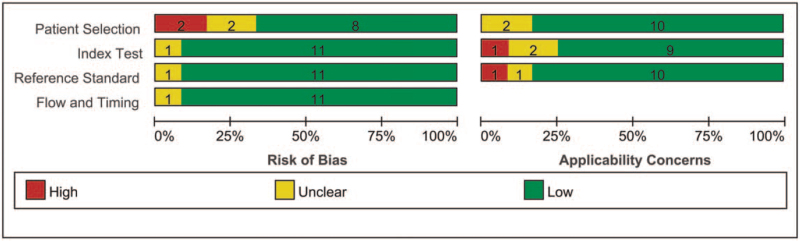
Overall methodological quality assessments of the included 12 articles based on QUADAS-2 tool. QUADAS-2 = quality assessment of diagnostic accuracy studies-2.

As shown in Figure 1, 370 articles were primarily obtained via the initial retrieval strategy. After screening the titles and abstracts, 333 articles were omitted because of unsuitable literary formats and unrelated study topics. Twenty-five articles were further omitted for duplicate studies or incomplete data after a thorough review of the manuscripts. Consequently, 12 articles were finalized for inclusion in this study.^[16,19–29]^ All the included articles fulfilled a relatively high score of ≥4 as per QUADAS-2, indicating that these studies were suitable for use in this meta-analysis (Table 1). Of the 12 articles included, quantitative real-time polymerase chain reaction assays on sputum^[26,29]^ (n = 2) and serum^[16,19–25,27–28]^ (n = 10) were carried out to detect the expression levels of miRNA-155 in 2 and 10 studies, respectively. Of the selected articles, 8 were based on the Han Chinese population,^[22–29]^ while the other 4 were based on populations in the Egypt, India, and Cameroon.^[16,19–21]^ In terms of age type, 4 articles focused on children with active TB,^[16,23–25]^ while 8 articles^[19–22,26–29]^ focused on adult active TB (Fig. 2).

**Table 1:** Characteristics of the included studies.

Author	Year	Country	Age	Sample (Case/control)	Specimen	Dysregulated status	Cutoff value	TP	FP	FN	TN	QUADAS-2
Abd-El-Fattah et al^[19]^	2013	Egypt	Adult	29/37	Serum	Up	Unclear	28	14	1	23	7
Cai et al^[24]^	2016	China	Child	30/30	Serum	Up	1.20	26	3	4	27	5
Cao et al^[25]^	2014	China	Child	40/60	Serum	Up	0.71	32	6	8	54	5
Chen et al^[26]^	2020	China	Adult	68/32	Sputum	Up	Unclear	64	2	4	30	6
Chen et al^[27]^	2017	China	Adult	100/45	Serum	Down	0.36	81	13	19	32	5
Kathirvel et al^[16]^	2020	India	Child	30/30	Serum	Up	0.70	28	1	2	29	7
Ndzi et al^[20]^	2019	Cameroon	Adult	83/42	Serum	Up	Unclear	66	21	17	21	6
Shi et al^[28]^	2019	China	Adult	72/45	Serum	Down	Unclear	30	7	42	38	7
Wagh et al^[21]^	2017	India	Adult	30/30	Serum	Down	Unclear	29	3	1	27	6
Wu et al^[22]^	2012	China	Adult	21/19	Serum	Up	Unclear	10	1	11	18	7
Zhou et al^[23]^	2016	China	Child	68/32	Serum	Up	Unclear	24	3	1	18	7
Ying et al^[29]^	2020	China	Adult	68/122	Sputum	Up	Unclear	64	15	4	107	7

FN = false-negative, FP = false-positive, QUADAS-2 = quality assessment of diagnostic accuracy studies-2, TN = true-negative, TP = true-positive.

### 3.2 Diagnostic accuracy of miRNA-155 in active tuberculosis

**Figure 3:**
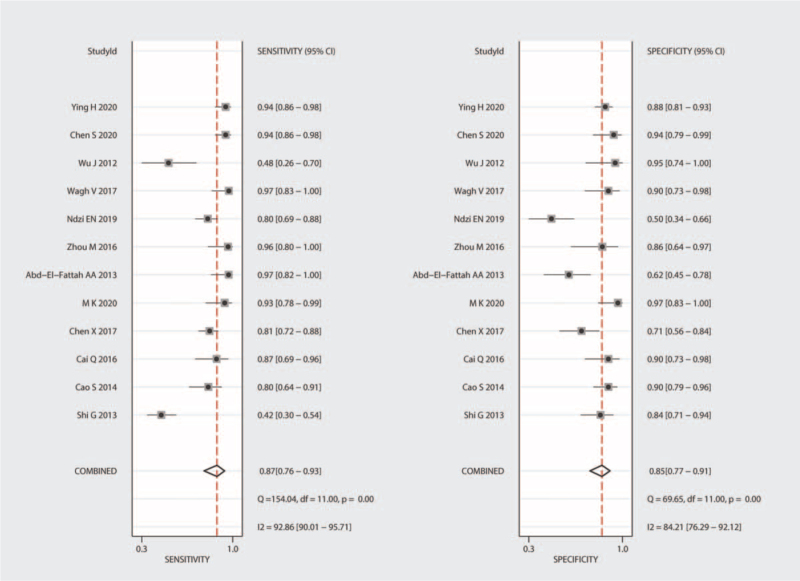
Forest plots of summary sensitivity and specificity of microRNA-155 in the diagnosis of active tuberculosis. CI = confidence interval.

**Figure 4:**
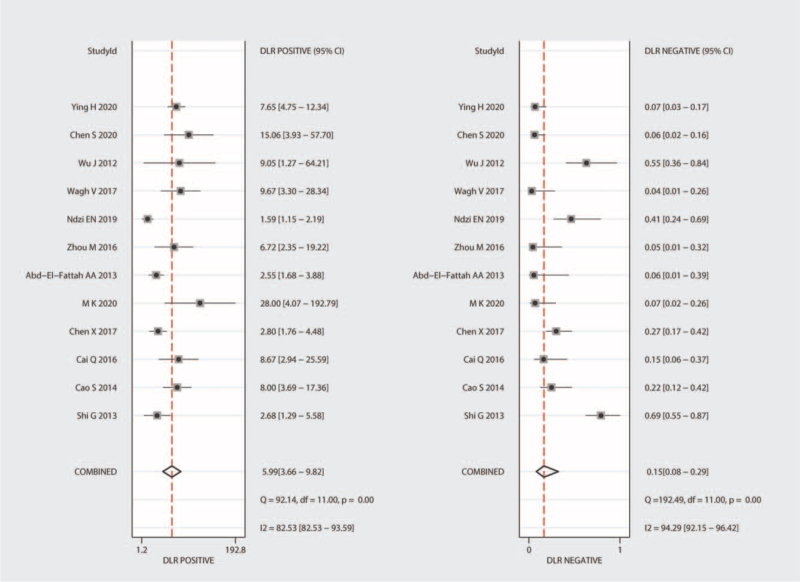
Forest plots of summary PLR and NLR of microRNA-155 in the diagnosis of active tuberculosis. CI = confidence interval, DLR = diagnostic likelihood ratio, NLR = negative likelihood ratio, PLR = positive likelihood ratio.

**Figure 5:**
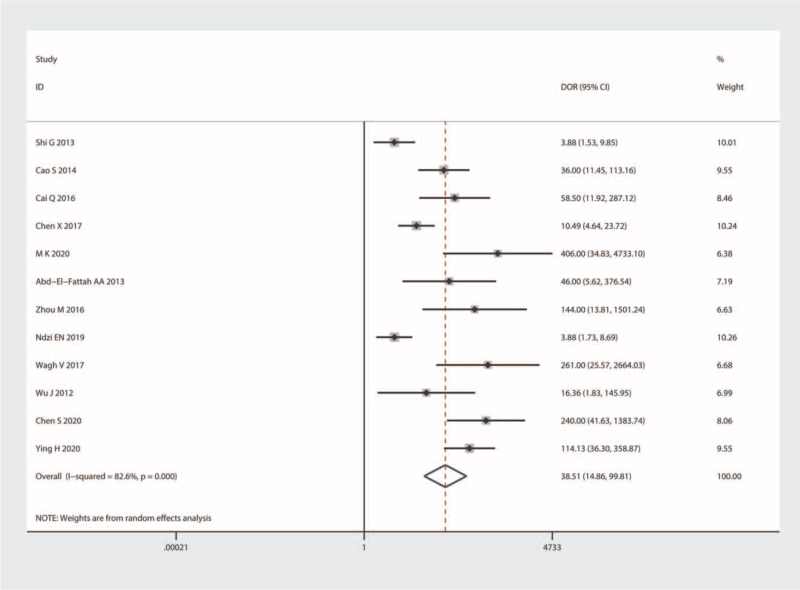
Forest plots of summary DOR of microRNA-155 in the diagnosis of active tuberculosis. CI = confidence interval, DOR = diagnostic ratio.

**Figure 6:**
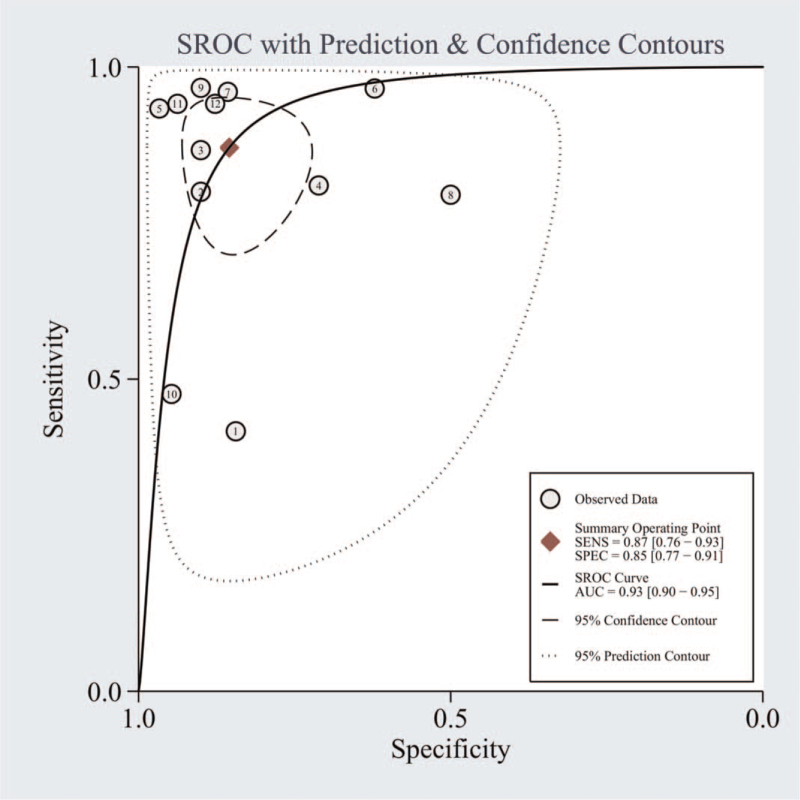
Summary ROC curves for microRNA-155 in the diagnosis of active tuberculosis. AUC = area under the curve, ROC = receiver operating characteristic.

Significant heterogeneity was observed in our results (I^2^ = 92.86% for SEN and I^2^ = 84.21% for SPE); hence, we selected the random-effects model for the next analysis. Forest plots of SEN and SPE are shown in Figure 3. The pooled SEN, SPE, PLR, and NLR were 0.87 (95% CI = 0.76–0.93), 0.85 (95% CI = 0.77–0.91), 5.99 (95% CI = 3.66–9.82), and 0.15 (95% CI = 0.08–0.29), respectively (Table 2 and Fig. 4), and the DOR was 38.51 (95% CI = 14.86–99.81) (Fig. 5). The AUC was 0.93 (95% CI = 0.90–0.95). Figure 6 shows the overall summary receiver operating characteristic curve. These results suggest that miRNA-155 could serve as a vital and adjuvant tool for the diagnosis of active TB.

**Table 2:** Subgroup analysis of the diagnostic efficacy of microRNA-155 in active tuberculosis.

Parameter	No. of studies	No. of patients	AUC	Sensitivity	Specificity	Heterogeneity	Meta-regression (*P* value)
Ethnicity							.59
Asian	8	467	0.90[0.88–0.93]	0.83[0.67–0.92]	0.87[0.82–0.91]	91%	
African	1	83	NA	NA	NA	NA	
Caucasian	3	89	0.95[0.92–0.97]	0.76[0.67–0.84]	0.90[0.82–0.95]	95%	
Specimen							.12
Serum	10	503	0.91[0.88–0.93]	0.85[0.71–0.93]	0.84[0.74–0.91]	95%	
Sputum	2	136	NA	NA	NA	NA	
Age							.22
Child	4	168	0.93[0.91–0.95]	0.89[0.80–0.94]	0.91[0.85–0.95]	0%	
Adult	8	471	0.90[0.87–0.92]	0.85[0.69–0.94]	0.82[0.70–0.90]	95%	
Dysregulated							.87
Upregulated	9	437	0.94[0.92–0.96]	0.89[0.79–0.94]	0.87[0.76–0.93]	89%	
Downregulated	3	202	0.89[0.86–0.93]	0.69[0.62–0.76]	0.81[0.73–0.87]	58%	
Overall	12	639	0.93[0.90–0.95]	0.87[0.76–0.93]	0.85[0.77–0.93]	95%	

AUC = area under the curve, NA = not available.

### 3.3 Threshold effect (TE) and SEA

As TE has been verified as the primary cause of heterogeneity in numerous meta-analysis studies,^[30]^ we assessed the TE of miRNA-155 using the Spearman correlation coefficient (−0.035, *P*-value = .913 [>.05]). The TE was absent in our meta-analysis. Thereafter, a SEA based on sample types, age, dysregulated status, and ethnicity was performed to explore heterogeneity. The pooled SEN, SPE, and AUC values for each subgroup are shown in Table 2. The statistical values for SEN, SPE, and AUC were similar in the Chinese population group (SEN = 0.83, SPE = 0.87, and AUC = 0.90), and the Caucasian population group (SEN = 0.76, SPE = 0.90, and AUC = 0.95). The results indicated that miRNA-155 was upregulated in most of the included studies. The analysis based on dysregulated status demonstrated that the upregulated group yielded a better DAE than the downregulated group. The pooled SEN, SPE, and AUC for the upregulated group were 0.89, 0.87, and 0.94, respectively, while the results for the downregulated group were 0.69, 0.81, and 0.89, respectively. It should be noted that the diagnostic efficacy of miRNA-155 in pediatric TB (AUC = 0.93, SEN = 0.89, SPE = 0.91) was higher than that in adult TB (AUC = 0.90, SEN = 0.85, SPE = 0.82).

### 3.4 Meta-regression and sensitive analysis

**Figure 7:**
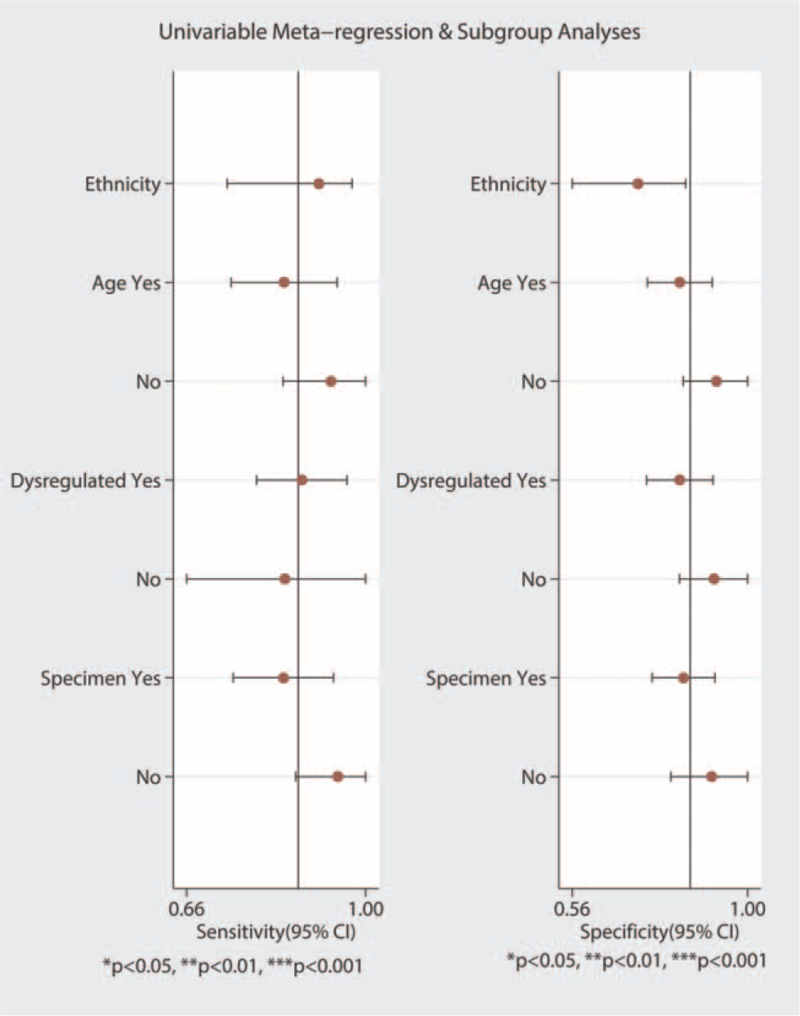
Univariable meta-regression and subgroup analyses. CI = confidence interval.

**Figure 8:**
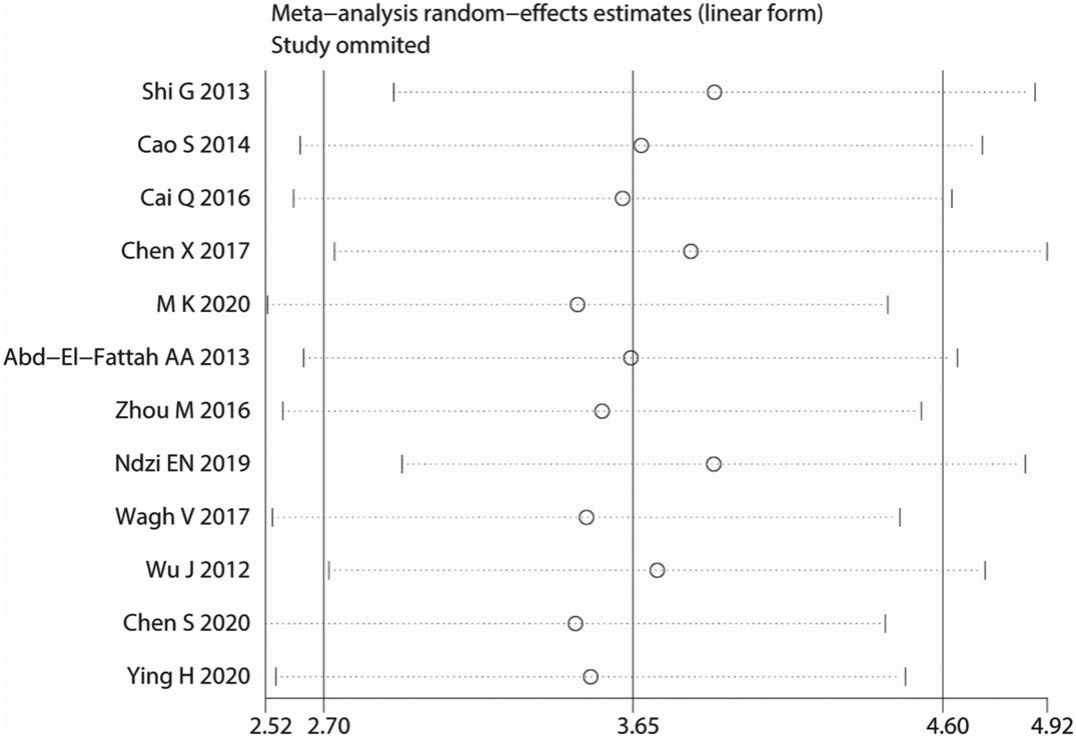
Sensitivity analysis of the overall pooled study.

The potential sources of heterogeneity were further explored through meta-regression analysis. Univariate meta-regression analysis showed that *P*-values with the covariates of ethnicity, sample type, dysregulated status, and age were 0.59, 0.12, 0.22, and 0.87, respectively (Fig. 7, Table 2). We also performed multivariable meta-regression analysis using the above-mentioned covariates and found that the covariates had no significant effect on heterogeneity. None of the individual studies were placed beyond the upper or lower CI limits, suggesting that the selected studies were homogeneously distributed (Fig. 8).

### 3.5 Publication bias and clinical utility of miRNA-155 in the diagnosis of active TB

**Figure 9:**
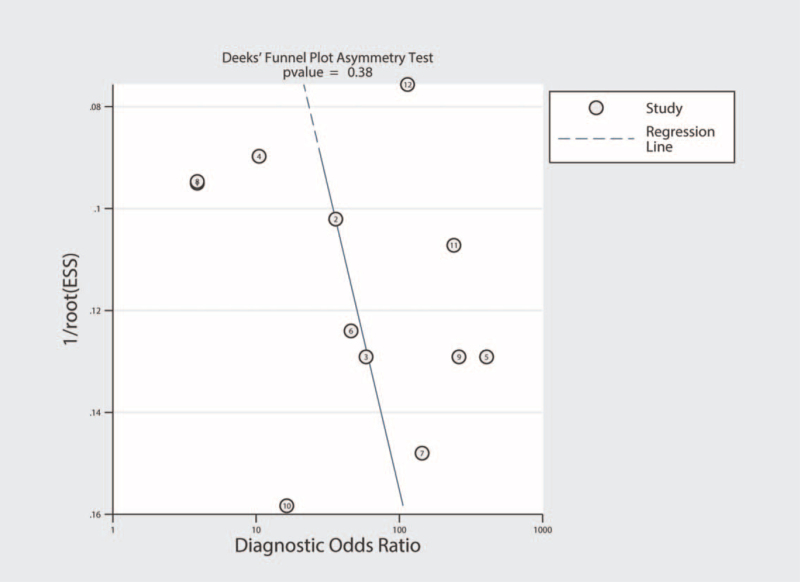
Deeks funnel plots for the assessment of publication bias.

**Figure 10:**
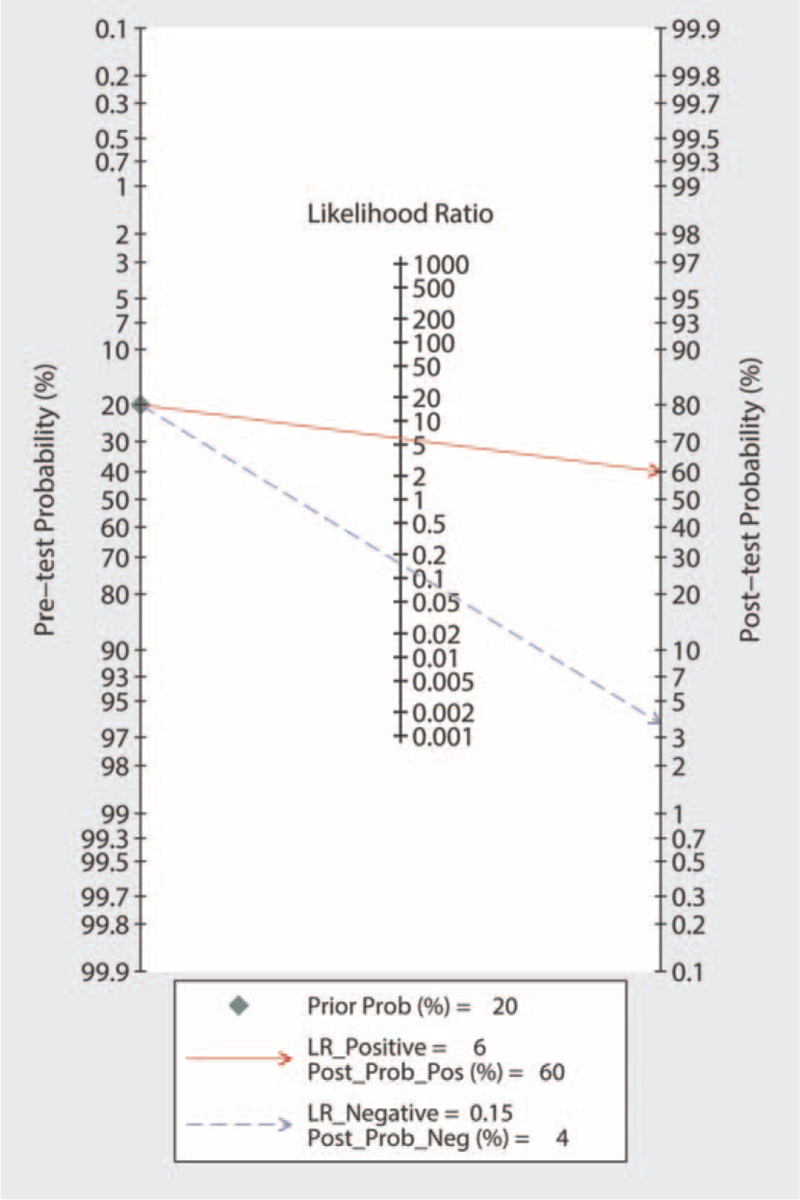
Fagan nomogram of microRNA-155 for diagnosis of active tuberculosis. LR = likelihood ratio.

Publication bias was investigated using the Deeks funnel plot asymmetry test. No significant publication bias was identified (*P* = .38) (Fig. 9). Fagan nomogram was used to describe the diagnostic value of miRNA-155 for active TB (Fig. 10).

## 4 Discussion

Despite recent technological developments in TB diagnosis, accurate and rapid diagnosis of active TB remains a clinical challenge. In recent years, with the application of RNA interference technology in cell growth, proliferation, differentiation, apoptosis, host immunity, and other biological processes, the regulatory mechanisms of miRNAs by mRNAs have been widely studied.^[31]^ miRNA expression varies between individuals and tissues, and specific miRNAs have been found to have an immunomodulatory effect on cells induced in response to MTB infection.^[15]^ miRNA-155 is commonly found in lung tissues and is involved in mediating immune-related processes such as hematopoiesis, tumors, and B-cell and T-cell gradings.^[32]^ By targeted regulation of mRNA and mediation of the expression of various cytokines, miRNA-155 may be involved in the development of lung diseases such as lung cancer, asthma, TB and pulmonary fibrosis.^[33]^ In recent years, miRNA-155 has been found to play an important role in TB infection. Wang et al^[34]^ found that miRNA-155 could promote cell autophagy and aid in the elimination of MTB from the body by regulating *RHEB* gene expression. They also demonstrated that miRNA-155 acts on *SHIP-1* to regulate the production of BCG-mediated reactive oxygen species. Some studies reported that the expression of miRNA-155 in MTB-infected RAW264.7 cells was significantly upregulated and closely related to the intracellular survival of MTB. MTB-induced miRNA-155 can subvert autophagic activities by targeting ATG3 in human dendritic cells.^[35,36]^ Cao et al^[25]^ reported that serum miRNA-155 levels in active TB patients were remarkably higher than those in healthy patients, suggesting that miRNA-155 is a biomarker for the diagnosis of active TB. All these studies show that miRNA-155 is an important regulator of TB infection by directly or indirectly affecting the expression of host genes.

However, owing to the different research designs and objects, the conclusions of studies on the role of miRNA-155 in TB are inconsistent. Wagh et al^[21]^ found that the expression of miRNA-155 in the serum of patients with pulmonary TB was downregulated compared to that in normal controls, and its diagnostic efficacy for active pulmonary TB needs to be further studied. Therefore, the purpose of the present study was to summarize the results obtained in different studies on this topic and search for common ground regarding the diagnostic efficacy of this miRNA. Our results showed that the combined SEN of miRNA-155 was 0.87, with SPE of 0.85, and AUC of 0.93, indicating that the overall diagnosis of active pulmonary TB using miRNA-155 has a moderate level of detection performance. A PLR value of 5.99 indicates that patients with active TB are approximately 6 times more likely to have a positive miRNA-155 test than healthy individuals. An NLR value of 0.15 means that only 15% of the negative PLR test results may be negative. Moreover, the DOR was 38.51, indicating that miRNA-155 possesses high discriminating accuracy for active pulmonary TB.

The DAE was similar in both the European descent and Chinese populations considered in this study, suggesting that the pathogenic effect of miRNA-155 for TB was similar in both populations. Nevertheless, the diagnostic efficacy of miRNA-155 was higher in children than in adults. Due to the rapid physical developmental processes occurring in children, their immune status and TB susceptibility differ from those of adults.^[23]^ However, the number of articles on the association between miRNA-155 and pediatric TB is relatively small. Therefore, the conclusions of this meta-analysis require further verification.

Exploring the source of heterogeneity is key to the meta-analysis. Since our results showed obvious heterogeneity, the source of this heterogeneity had to be clarified. The Spearman correlation coefficients for this study were −0.035 and 0.913, which indicate that TE was not the cause of heterogeneity. SEN analysis was then used to test whether this heterogeneity originated from a single study, and the results showed that the selected studies were uniformly distributed. Other factors causing heterogeneity were analyzed using subgroup and meta-regression analyses. We verified 4 covariates, including ethnicity, sample type, dysregulated status, and age. These factors were found to be unrelated to the heterogeneity in the study. The heterogeneity may be related to the study design, experimental methods used to analyze miRNA-155, or the severity of the disease, but these detailed data factors have not been mentioned in the literature. Hence, the source of heterogeneity in this study could not be determined. The Deeks funnel chart revealed no significant publication bias in the diagnostic meta-analysis of miRNA-155.

Our meta-analysis has several limitations. First, most of the studies eligible for inclusion in our analysis were from China, and the meta-analysis considered only Chinese, Caucasian and African populations. Of these, only 1 study examined the association between miRNA-155 expression and TB diagnosis in an African population. Second, among the 12 articles selected, the sample size of active pulmonary TB was only 639. Due to the small sample size, the subgroup analysis of the included studies was limited. Third, the heterogeneity in the study could not be adequately explained with the results of the meta-regression and SEN analyses.

In conclusion, our meta-analysis demonstrated the potential of miRNA-155 in distinguishing patients with active TB from healthy controls, especially in children. Therefore, miRNA-155 may be a highly accurate diagnostic tool for active TB. Nevertheless, further large-scale prospective studies are warranted to validate the conclusions of this study before the clinical use of miRNA-155 as a diagnostic marker.

## Author contributions

**Conceptualization:** Jie He.

**Data curation:** Xiaoyan Li, Jie He.

**Methodology:** Xiaoyan Li, Guodong Wang.

**Validation:** Jie He, Jian Sun.

**Writing – original draft:** Xiaoyan Li, Jie He.

**Writing – review & editing:** Xiaoyan Li, Jie He.
